# Should We Abandon the *t*-Test in the Analysis of Gene Expression Microarray Data: A Comparison of Variance Modeling Strategies

**DOI:** 10.1371/journal.pone.0012336

**Published:** 2010-09-03

**Authors:** Marine Jeanmougin, Aurelien de Reynies, Laetitia Marisa, Caroline Paccard, Gregory Nuel, Mickael Guedj

**Affiliations:** 1 Programme Cartes d'Identité des Tumeurs (CIT), Ligue Nationale Contre le Cancer, Paris, France; 2 Department of Biostatistics, Pharnext, Paris, France; 3 Department of Applied Mathematics (MAP5) UMR CNRS 8145, Paris Descartes University, Paris, France; 4 Statistics and Genome Laboratory UMR CNRS 8071, University of Evry, Evry, France; University of Michigan, United States of America

## Abstract

High-throughput post-genomic studies are now routinely and promisingly investigated in biological and biomedical research. The main statistical approach to select genes differentially expressed between two groups is to apply a *t*-test, which is subject of criticism in the literature. Numerous alternatives have been developed based on different and innovative variance modeling strategies. However, a critical issue is that selecting a different test usually leads to a different gene list. In this context and given the current tendency to apply the *t*-test, identifying the most efficient approach in practice remains crucial. To provide elements to answer, we conduct a comparison of eight tests representative of variance modeling strategies in gene expression data: Welch's *t*-test, ANOVA [1], Wilcoxon's test, SAM [2], RVM [3], limma [4], VarMixt [5] and SMVar [6]. Our comparison process relies on four steps (gene list analysis, simulations, spike-in data and re-sampling) to formulate comprehensive and robust conclusions about test performance, in terms of statistical power, false-positive rate, execution time and ease of use. Our results raise concerns about the ability of some methods to control the expected number of false positives at a desirable level. Besides, two tests (limma and VarMixt) show significant improvement compared to the *t*-test, in particular to deal with small sample sizes. In addition limma presents several practical advantages, so we advocate its application to analyze gene expression data.

## Introduction

During the last decade, advances in Molecular Biology and substantial improvements in microarray technology have led biologists toward high-throughput genomic studies. In particular, the simultaneous measurement of the expression levels of tens of thousands of genes has become a mainstay of biological and biomedical research.

The use of microarrays to discover genes differentially expressed between two or more groups (patients *versus* controls for instance) has found many applications. These include the identification of disease biomarkers that may be important in the diagnosis of the different types and subtypes of diseases, with several implications in terms of prognostic and therapy [7], [8].

A first approach to identify differentially expressed genes is known as the Fold-Change estimation (FC). It evaluates the average log-ratio between two groups and considers as differentially expressed all genes that differ by more than an arbitrary cut-off. So defined, FC lacks of a solid statistical footing [9]: it does not take the variance of the samples into account. This point is especially problematic since variability in gene expression measurements is partially gene-specific, even after the variance has been stabilized by data transformation [10], [11].

Rather than applying a FC cutoff, one should prefer statistical tests: they standardize differential expression by considering their variance [9], [12]. Furthermore, corresponding effect sizes, confidence intervals and *p*-values are essential information for the control of false-positives [13] and meta-analysis [14].

The *t*-test is certainly the most popular test and has been matter of discussion. Computing a *t*-statistic can be problematic because the variance estimates can be skewed by genes having a very low variance. These genes are associated to a large *t*-statistic and falsely selected as differentially expressed [2]. Another drawback comes from its application on small sample sizes which implies low statistical power [12]. Consequently, the efficacy of a *t*-test along with the importance of variance modeling have been seriously called into question [15]. It has led to the development of many innovative alternatives, with hope of improved variance estimation accuracy and power.

These alternatives appear very diverse at a first sight, but fall into few nested categories relying on both statistical and biological hypotheses: parametric or non-parametric modeling, frequentist or Bayesian framework, homoscedastic hypothesis (same variance between groups of samples) and gene-by-gene variance estimation. Further propositions come from the field of machine-learning for instance [16], but lie beyond the scope of our study.

A disadvantage of having so many alternatives is that selecting a different test usually identifies a different list of significant genes since each strategy operates under specific assumptions [17]. Moreover, despite the wealth of available methods, the *t*-test remains widely used in gene-expression studies, presumably because of its simplicity and interpretability. Given the tendency to use this method, identifying which approach is the most appropriate to analyze gene expression data remains a crucial issue. Nevertheless, if the development of new methodologies is still an active topic of publication, only few studies have addressed their comparison. This is probably due to the difficulty to implement a realistic framework of comparison for which the differentially expressed genes are known in advance.

In order to sidestep many problems, comparisons frequently rely on the analysis of gene lists resulting from the application of several methods [18] and simulations for which truly differentially expressed genes are known [6]. More empirical alternatives include the use of re-sampling methods (to compare genes from small subsets of samples and those from the full dataset) [3], [19], and the use of spike-in data for which a set of genes are differentially expressed by design [12], [20]. Finally Jeffery et al. [18] explore an indirect approach by assessing classification performance obtained with genes resulting from the application of the methods to compare. The heterogeneity of the strategies adopted in the literature and the diversity of tests investigated make the formulation of general conclusions difficult. In addition, to our knowledge, no study has focused on the direct comparison of a wide range of variance modeling strategies.

Consequently, we conduct a comparison study of eight tests representative of variance modeling strategies in gene expression data: Welch's *t*-test, ANOVA [1], Wilcoxon's test, SAM [2], RVM [3], limma [4], VarMixt [5] and SMVar [6]. The comparison process relies on four steps: gene list analysis, simulations, spike-in data and re-sampling. Our aim is to benefit from the specificity of each strategy, to make our results comparable to previous studies and to ease the formulation of general, robust and reproducible conclusions.

So defined, we follow a standard statistical framework. First, our main focus concerns the issue of data reduction which relies on the form of the test statistic and impact directly the resulting power. A separate but important issue is calibration (i.e. the accuracy of *p*-values) which can impact the false-positive rate (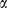). So at each step of the process, tests are compared in terms of statistical power assessed at the same false-positive rate. Control of the false-positive rate to the desired value is checked for each test which is, to our opinion, too rarely considered in the literature. Eventually, in addition to an efficacy comparison, we find relevant to confront each test in terms of practical consideration such as execution time and ease of use.

## Methods

### Statistical background

Differential analysis consists in testing the null hypothesis (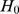) that the expected values of expression for a given gene are equal between two groups of interest (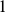 and 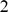), against the alternative hypothesis (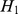) that they differ. Let 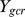 the level of expression observed for gene 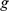, replicate 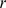, under group 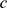; the general model is then given by:
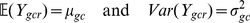
So defined, the null hypothesis to test comes down to:
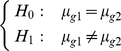
Given a statistical test, type-I error-rate 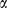 (resp. type-II error-rate 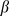) commonly refers to the probability to reject (resp. accept) 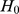, 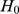 being true (resp. false). The statistical power of the test is then defined as the ability to reject 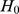 when it is actually false:
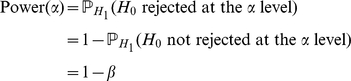



Type-I and II errors are inversely related: the smaller the risk of one, the higher the risk of the other. Consequently the power depends directly on 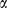, and a valid comparison of several tests has to be driven at the same type-I error-rate to overcome the issue of calibration.

The type-I error-rate is often referred to as false-positive rate. It differs from the false-discovery rate (FDR) in the sense that it represents the rate that truly null features are called significant whereas the FDR is the rate that significant features are truly null [21].

### Selection of the eight tests

This selection has focused on tests broadly applied in the literature and representative of different variance modeling strategies. The eight tests selected are described in detail in Methods S1 and re-implemented in R to simplify their application. The package is available on demand.

Briefly, most of the eight tests are parametric and estimate a gene-by-gene variance: ANOVA (homoscedastic), Welch's *t*-test (heteroscedastic), RVM (homoscedastic), limma (homoscedastic and based on a Bayesian framework) and SMVar (heteroscedastic and based on structural model); we also select two non-parametric approaches with the Wilcoxon's test and the SAM test, which do not rely on assumptions that the data are drawn from a given probability distribution.

Besides, variances estimated on a set of genes are thought to lead to an undesirable amount of false-positives. Attributing a common variance to all the genes is clearly not a solution, even when sample sizes are small. Several proposals make the assumption that genes with the same expression level have approximatively the same variance [22], [23]. However this is not realistic and also leads to false-positives [24]. We find VarMixt more subtle: it makes the assumption that classes of genes can be identified based on similar response to the various sources of variability (mixture model); the variance of each homogeneous class is then accurately estimated from a large set of observations; the individual gene variance is then replaced by its “class” variance.

### Comparison process

#### Gene list analysis

An intuitive first step to compare the tests is to investigate the consistency between gene lists resulting from the application of each test on real data. Here we apply this approach to five publicly available data sets (Table 1) to assess the overlap between gene lists and to identify similar behaviors among the variance modeling strategies.

**Table 1:** Data sets used for the gene list analysis.

Data-set	Groups	Sample size	Publication
Lymphoid tumors	Disease staging	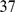	Lamant et al. 2007 [26]
Liver tumors	TP53 mutation	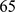	Boyault et al. 2007 [27]
Head and neck tumors	Gender	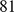	Rickman et al. 2008 [28]
Leukemia	Gender	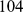	Soulier et al. 2006 [29]
Breast tumors	ESR1 expression	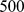	Bertheau et al. 2007 [30]

The five data sets come from the *Cartes d'Identité des Tumeurs* (CIT, http://cit.ligue-cancer.net) program and are publicly available. All the microarrays are Affymetrix U133A microarrays with 22,283 genes.

In addition to the eight tests, we define a “control” test that draws for each gene a *p*-value from a Uniform distribution between 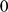 and 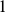. Then, we applied the tests to the five data-sets to identify gene differentially expressed by setting a *p*-value threshold of 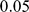.

Gene list similarities between tests are analyzed and visualized using a Hierarchical Clustering (binary metric and the Ward's aggregation algorithm, R package *stats*) and Principal Component Analysis (R package *ade4* [25]). For more details please refer to Methods S1 and Table S1.

#### Simulation study

The purpose of simulations is to estimate power and false-positive rate on a large range of simulated data sets, in order to compare the tests under simple and sometimes extreme situations. We define a reference model (denoted 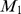), frequently adopted in the literature and that matches the assumptions of the *t*-test. Under 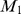, gene expressions for the groups 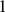 and 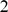 are drawn from Gaussian distributions of same variance (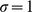):
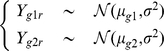
Under 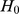: 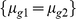 while under 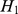: 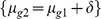, with 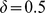.

Then, we propose three extensions of 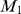 (denoted 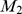, 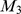 and 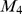) designed to be less to the *t*-test advantage. 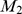 is quite similar but expression levels are now drawn from a Uniform distribution of same parameters. 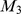 applies a mixture model on variances and corresponds to the VarMixt hypothesis; genes are then divided into three classes of variance. Under 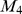, 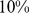 of the genes are simulated with small variances (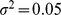) since they can lead to an increase of false-positive rate when the *t*-test is applied.

**Figure 1:**
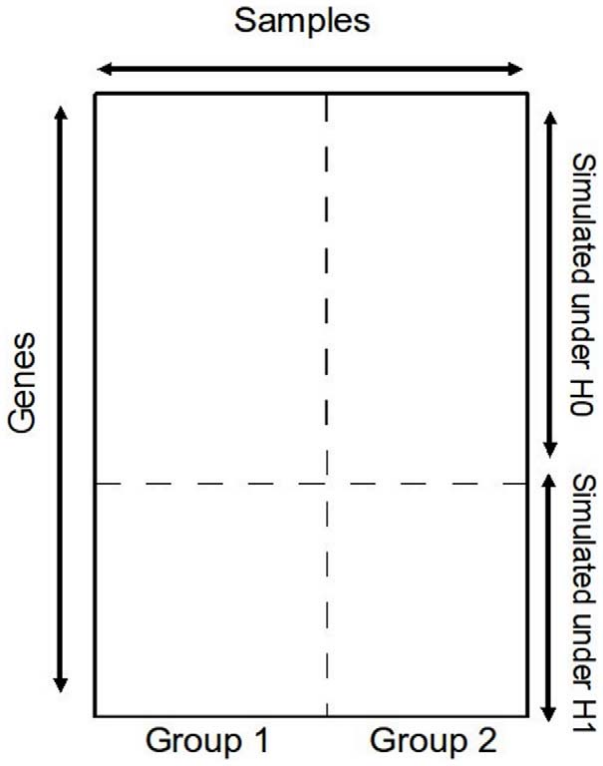
Data matrix resulting from simulations. Rows refer to genes simulated under 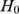 and 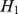, columns refer to samples of both groups to compare.

For each model we simulate 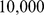 independent genes under 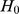 to assess the false-positive rate attached to each test, and 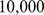 under 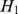 to compute their respective power. False-positive rate and power are both assessed at a *p*-value threshold of 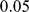. Sample size ranges from 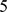 to 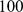 samples per group. The simulated data matrix is given Figure 1.

#### Spike-in data set

The Human Genome U133 data set is used to test and validate microarray analysis methods (http://www.affymetrix.com). The data set consists in 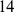 hybridizations of 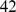 spiked transcripts in a complex human background at concentrations ranging from 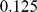 pM to 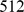 pM. Each group includes three replicates. We perform the 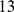 pairwise comparisons for which “spike-in” genes have a true fold-change of two [5].

The whole data set contains 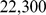 genes. The 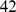 spike-in genes are designed to be differentially expressed (under 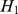) and used for power estimation. To be able to compute the false-positive rate, the 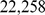 remaining genes are forced to be under 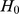 by permutation of the group labels. False-positive rate and power are both assessed at a *p*-value threshold of 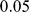.

#### Re-sampling approach

The main idea is to assess the ability of a test to select from small subsets of samples (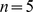 and 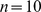), genes determined as differentially expressed from the full data set. The strategy can be summarized in four steps:

Step 1: From the 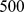 samples data set (Table 1) split into two groups to compare, we define a set of differentially expressed genes (*p*-value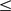 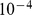 with the Welch's *t*-test). This set is considered in Step 3 as the “truth” to estimate power.

Step 2: 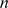 samples are drawn from each group and the eight tests are performed on this subset of the initial data. We apply the Benjamini and Hochberg correction at a 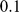 FDR level [31].

Step 3: From Step 2 we estimate power as the proportion of genes defined as differentially expressed at Step 1 and detected at Step 2.

Step 4: Steps 2 and 3 are iterated 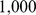 times. Finally power is averaged over the 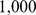 iterations.

## Results

### Gene list analysis

**Figure 2:**
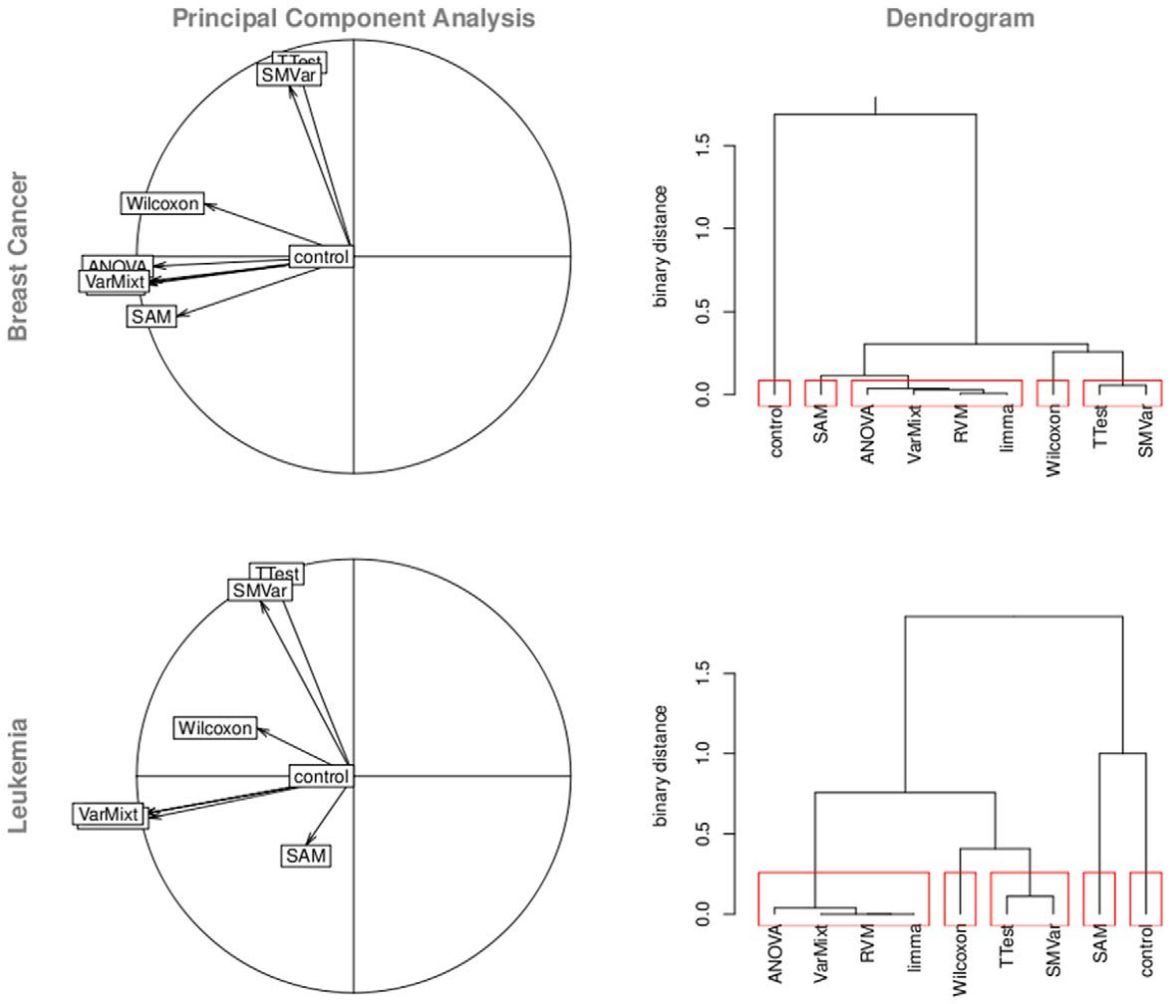
Gene list analysis. PCAs and dendrograms are generated based on the gene lists resulting from the application of the eight tests of interest and the control-test. Here we show results for two data sets comparing ESR1 expression in breast cancer and gender in leukemia. Both outline five clusters of tests.

Figure 2 represents PCAs and dendrograms resulting from gene list analysis. The cumulative inertia explained by the two first axes of PCA is about 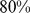. Both representations underline the same tendencies.

As expected, gene lists resulting from the control-test are clearly independent from the other ones, since it selects genes (differentially expressed or not) uniformly. Then, the eight tests show various behaviors. Six tests clusterize in two distinct groups: {*t*-test; SMVar} and {VarMixt; limma; RVM; ANOVA}. The proportion of common genes selected by two tests of the same cluster is about 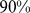. On the other hand, Wilcoxon and SAM do not clearly fall in one of the two main groups: Wilcoxon tends to consistently lie between them, whereas SAM does not present a reproducible behavior.

To summarize, homoscedastic (VarMixt, limma, RVM and ANOVA), heteroscedastic (*t*-test and SMVar) variance modeling strategies are well discriminated by a similarity analysis of gene lists. It outlines the interesting property that similar modeling strategies in theory imply similar results in practice.

### Simulation study

**Figure 3:**
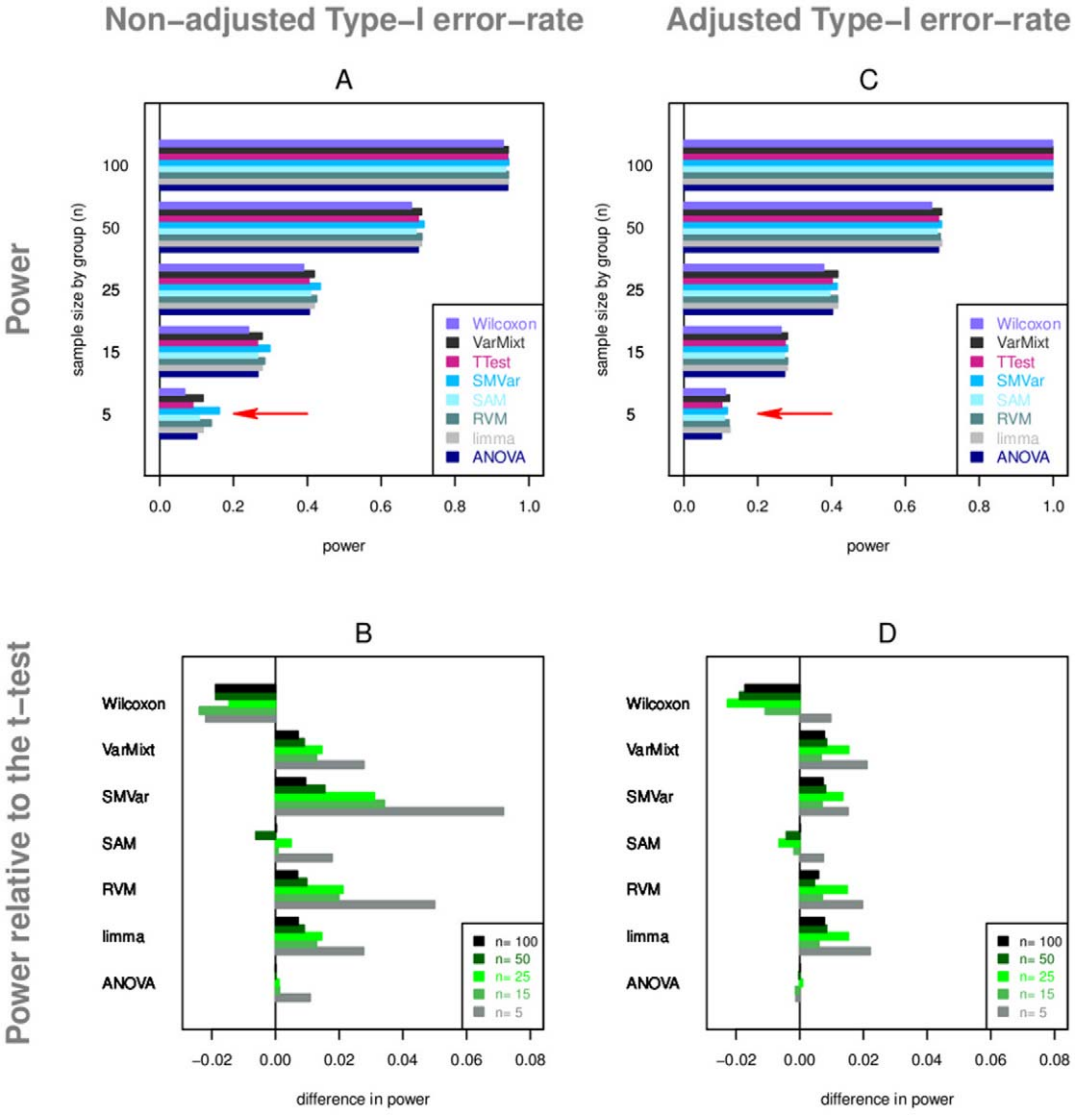
Power study from simulations (Gaussian model, M1). Power values are calculated at the 5% level and displayed according to the sample size. Figures A and C represent power values. Red arrows highlight the effect of false-positive rate adjustment on power values. Figures B and D represent power values relative to t-test. Figures A and B concern power values calculated at the actual false-positive rate. Figures C and D concern power values calculated at the adjusted false-positive rate.

First, we evaluate power according to sample size under the simulation model 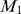 (Figure 3). On Figure 3-A, we notice little difference between the tests (less than 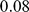), particularly for large samples as expected. Wilcoxon is not as good as the other tests in most cases. SAM and ANOVA show equivalent performance to the *t*-test. VarMixt, RVM and limma tend to provide an increase in power, and SMVar slightly outperforms all the tests (Figures 3-A and B).

As we know, these preliminary results are valid only if all the tests meet the theoretical 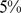 false-positive rate when applying a *p*-value threshold of 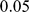. Table 2 gives the observed false-positive rate for each test under small and large sample sizes and sheds light on the fact that some tests clearly deviate from the 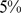 level and return biased *p*-values. Observed deviations are more accentuated for small sample sizes compared to large ones. SMVar and RVM inflate the expected number of false-positives whereas Wilcoxon and the *t*-test tend to be conservative; ANOVA, SAM, limma and VarMixt show no deviation.

**Table 2:** False-positive rate study from simulations.

	M1		M2		M3		M4	
Sample size	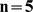	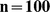	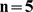	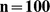	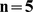	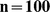	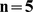	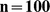
*t*-test^▾^	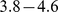	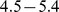	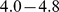	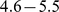	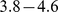	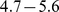	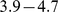	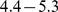
ANOVA	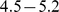	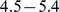	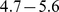	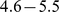	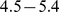	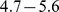	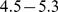	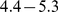
Wilcoxon^▾^	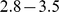	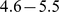	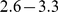	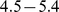	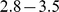	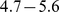	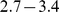	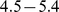
SAM	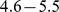	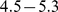	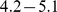	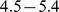	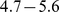	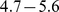	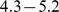	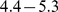
RVM^▴^	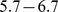	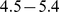	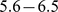	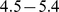	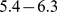	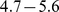	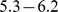	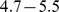
limma	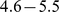	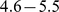	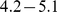	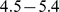	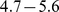	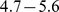	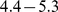	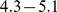
SMVar^▴^	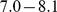	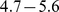	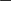	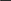	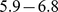	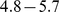	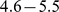	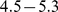
VarMixt	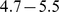	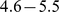	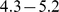	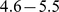	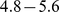	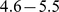	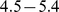	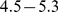

For small and large samples, this table presents the 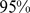 confidence-interval of false-positive rate obtained by applying a threshold of 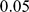 to the *p*-values. Up triangles ▴ (resp. down triangles ▾) indicate an increase (resp. a decrease) of the false-positive rate compared to the expected level of 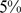. Two triangles inform of a deviation in both small and large sample sizes.

Regarding these observations, the tests inefficient to control the false-positive rate at the expected 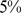 level have to be adjusted by a time consuming Monte-Carlo procedure. Figures 3-C and D present power results at adjusted and hence valid false-positive rates. Differences are clearly reduced compared to Figures 3-A and B which confirms that part of the difference in power observed is due to actual difference in false-positive rate, particularly concerning SMVar. After adjustment VarMixt, RVM and limma tend to be the best tests although they provide an insignificant gain compared to the *t*-test; Wilcoxon remains the less powerful. ANOVA has performance comparable to the *t*-test which is interesting: under the same variance between the two groups, tests that make the corresponding homoscedastic assumption (ANOVA) do not show improved power compared to heteroscedastic ones (Welch *t*-test).

Surprisingly, model 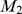 leads to the same conclusions (data not shown). Here expression values follow a Uniform distribution instead of a Gaussian one, which does not match the assumption of parametric approaches. Compared to model 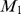, we were expecting to note a more striking increase in power for Wilcoxon, which is not observed. This result confirms that *t*-test and assimilated approaches are quite robust to the Gaussian assumption. Indeed the Central Limit Theorem implies that even if expression values are not Gaussian, the *t*-statistic resulting from the comparison of two groups is likely to be. It should be noticed that the structural model of SMVar is not able to provide results for the uniform model.

Finally models 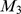 and 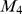 also lead to the same conclusions, with an overall loss of power (data not shown).

### Spike-in data set

**Figure 4:**
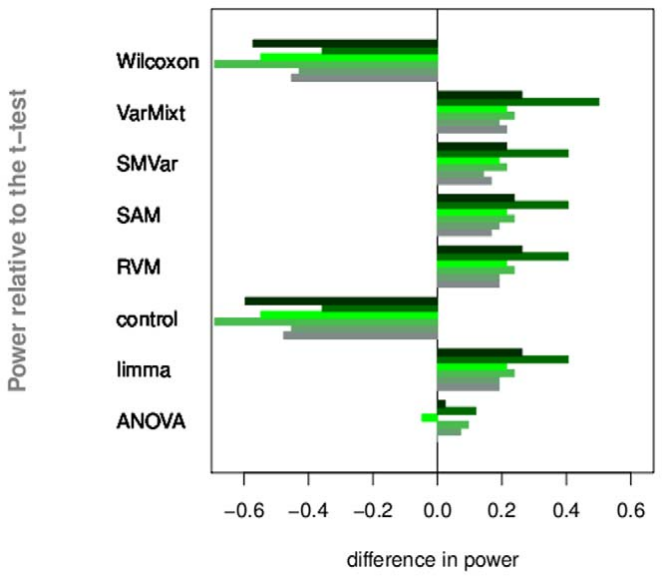
Spike-in data set. Power values are calculated at the 5% level and displayed according to six of the 13 pairwise comparisons.

Spike-in data confirm observations and conclusions made on the simulations. SMVar and RVM inflate the expected number of false-positives whereas Wilcoxon and the *t*-test tend to be conservative. Power values adjusted to a valid false-positive rate present more significant differences than in simulations (Figure 4): with an average decrease of almost 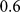, Wilcoxon is the less powerful and similar to the “control” test; ANOVA shows equivalent performance than the *t*-test; VarMixt, RVM, SMVar and limma provide a significant increase in power with an average gain of 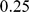. With performance comparable to the best tests, SAM has a different behavior than in simulations.

### Re-sampling approach

**Figure 5:**
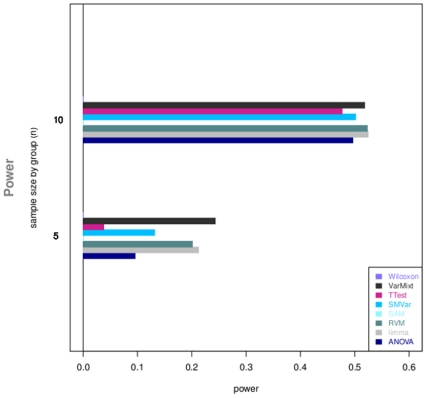
Re-sampling approach. Power values are calculated at a 0.1 FDR level and displayed according to the sample size.

This approach corroborates tendencies obtained with simulations and spike-in data (Figure 5): limma, VarMixt and RVM perform much better than other tests in identifying differentially expressed genes, while SMVar is somewhat less efficient than the three top-tests. ANOVA and the *t*-test still show equivalent performance, although ANOVA presents here a slight but significant improvement.

Wilcoxon and SAM were never able to detect genes determined as differentially expressed. Indeed the calibration performed can not reach *p*-value lower than 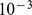 for small sample sizes. After the Benjamini-Hochberg correction at a 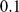 FDR level (corresponding here to a 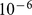 *p*-value threshold), they do not detect any gene as differentially expressed.

### Practical comparison

Concerning time of execution and ease of use, the *t*-test and ANOVA are the most efficient as they rely on standard statistical considerations and have benefited of improved implementations. On real high-throughput data, both take few seconds to treat tens of thousands of genes. In terms of time of execution, limma appears as efficient as the *t*-test and ANOVA, which is a noteworthy point. SMVar, RVM and SAM run in longer but still reasonable time (up to 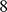 minutes in our case). Varmixt turns out to be the slowest approach (up to 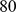 minutes) as it relies on a time consuming EM algorithm.

## Discussion

Given the current tendency to apply the *t*-test to gene expression data and the wealth of available alternatives, finding the most appropriate approach to handle differential analysis is critical.

To address this problematic and provide some answers, we develop a comparison process of eight tests for differential expression. It is based on gene list analysis, simulations, spike-in data and re-sampling, with the intention to benefit from the specificity and advantages of each strategy.

Gene list analysis do not properly compare test performance and hence lead to limited conclusions. However it is an appropriate preliminary approach that focuses on similarities between test results. An analysis of the consistency between gene lists outlines general tendencies that can help in interpreting differential analysis results. In our case, we observed comparable results between tests based on similar variance modeling strategies.

The three other approaches (simulations, spike-in data and re-sampling) propose a direct comparison of power values. Simulations represent a convenient statistical framework as genes under 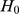 and 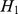 are known in advance. In addition different hypotheses on data structure can be specified under different simulation models. Here, the three further models (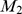, 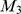 and 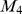) lead actually to the same conclusions than the reference Gaussian one (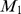). If simulations do not allow to observe significant differences in power between the tests, they still reveal reproducible tendencies. In addition, simulations turn out to be the gold standard to check possible deviations from the expected false-positive rate. However it is unclear whether simulated data sets can sufficiently and realistically reflect the noise inherent in real microarray data [32].

More empirical alternatives include the use of spike-in data and re-sampling. Spike-in genes can represent gene expression better than simulations. In our case it confirms conclusions from simulations with more significant differences in power. Regarding the Affymetrix data set we used, a criticism of this approach could be that the small number of actual spike-in genes does not allow a very accurate power estimation. Moreover variation across technical replicates is likely to be lower than that typically observed across true biological replicates, and many biological effects of interest may be smaller than two-fold [12].

In this context, a re-sampling approach takes advantage of the complexity found in real data. Differentially expressed genes are not known but determined from a large data set (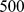 samples in our case); power is then evaluated on a subset of the data. Results are comparable to those obtained with simulations and spike-in data. However this approach can be considered as limited in that it assumes that gene lists generated on the full dataset are correct; besides it is fastidious to implement and extremely time consuming.

By applying four distinct comparison strategies with specific advantages and drawbacks: **(i)** we ensure to offset the limitations of each strategy and **(ii)** we provide robust conclusions on test performance.

We applied this comparison process to eight tests representative of different variance modeling strategies. Results are summarized in Table 3. A first important result concerns the control of the false-positive rate, which is often disregarded in the literature. Under 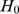, distribution of *p*-values is supposed to be uniform and the false-positive rate resulting from a *p*-value threshold of 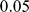 should be controlled at 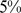. Deviation from this major assumption may indicate biased *p*-values. In both simulations and spike-in data, some tests deviate from the expected false-positive rate, which partly explains some differences in power (namely SMVar, RVM and Wilcoxon). For the purpose of our study, we performed a Monte-Carlo based adjustment of the false-positive rate to formulate comparable conclusions across all the tests. However in practice this adjustment remains fastidious to implement. In consequence, we strongly advocate to avoid using these tests until a proper corrected version is made available.

**Table 3:** Summary table.

	False-positive rate		Power		In practice	
	Small samples	Large samples	Small samples	Large samples	Ease of use	Execution time
**t-test**	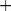	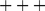	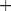	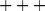	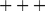	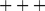
**ANOVA**	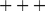	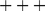	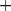	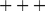	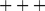	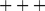
**Wilcoxon**	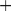	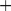	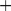	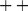	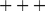	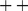
**SAM**	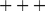	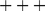	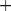	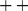	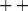	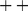
**RVM**	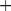	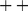	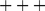	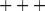	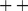	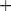
**limma**	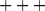	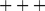	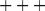	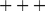	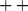	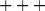
**VarMixt**	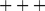	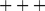	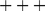	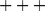	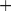	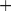
**SMVar**	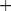	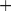	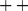	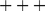	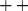	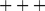

This table summarizes the results of our study in terms of false-positive rate, power and practical criteria. The number of “+” indicates the performance, from weak (+), to very good one (+++).

Overall, Wilcoxon and SAM show weak performance. One of our simulation model (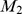) clearly outlines the robustness of parametric tests to the Gaussian assumption. Concerning SAM, our results do not allow to formulate clear conclusions and reflect existing doubts about its efficacy [18], [33].

Compared to the *t*-test, limma and VarMixt consistently show real improvement, in particular on small sample sizes. Limma has often been discussed in the biostatistical field and its good performance has been reported [12], [18], [24]. Surprisingly VarMixt does not appear as weak as similar methods evaluated by Kooperberg et al. [24]. Presumably it benefits from a more realistic mixture model on variances, less likely to generate false-positives.

If limma and VarMixt are equivalent regarding both power and false-positive rate, in practice limma presents several further advantages in terms of execution time. In addition, limma can be generalized to more than two groups which makes it relevant to many broader situations.

To conclude, we have developed a comprehensive process to compare statistical tests dedicated to differential analysis. This approach can be used as the basis to evaluate performance of methods developed in the near future. In addition, to answer our initial question “Should we abandon the *t*-test”, limma provides a substantial improvement compared to the *t*-test, particularly for small samples. However the *t*-test remains easy to apply through a wide-range of genomic analysis tools whereas limma can appear more difficult to implement at a first sight. To promote its application we make available on demand a simplified R version of limma dedicated to the analysis of two groups of samples.

## Supporting Information

Methods S1 A detailed description of (i) the eight tests included in the study and (ii) the gene list analysis process.
(0.09 MB PDF)

Table S1 Example of binary matrix. For a given test, the genes identified as differentially expressed (“1”) and not differentially expressed (“0”) at a given p-value threshold are reported in the binary matrix.
(0.01 MB PDF)
