# Supplementary material for: Should We Abandon the t-Test in the Analysis of Gene Expression Microarray Data: A Comparison of Variance Modeling Strategies
Source: PLoS One. 2010 Sep 3;5(9):e12336. doi: 10.1371/journal.pone.0012336 (PMC2933223; doi:10.1371/journal.pone.0012336)
Supplement: Methods S1 — A detailed description of (i) the eight tests included in the study and (ii) the gene list analysis process. (0.09 MB PDF) [file pone.0012336.s001.pdf]

Supporting Information

Contents

1 Test description **1**
1.1 Parametric tests 1
1.2 Non-parametric tests 4

2 Gene list analysis **5**
2.1 Hierarchical Clustering 5
2.2 Principal Component Analysis 5

1 Test description

In this section, we will describe the various tests mentioned in the article. Our main focus is on variance modelling.
We will distinguish parametric tests (that assume data come from a type of probability distribution) of non-parametric tests (that are distribution free). In the following, we will consider binary tests, i.e. comparison of two groups ($c = 1, 2$).

1.1 Parametric tests

Let Y_{gcr} the level of expression observed for gene g, replicate r, under group c; we assume that:

$$\mathbb{E}(Y_{gcr}) = \mu_{gc} \quad \text{and} \quad \mathbb{V}(Y_{gcr}) = \sigma^2_{gc}$$

The estimators of the parameters μ_{gc} and σ_{gc} are given by $\hat{\mu}_{gc}$ and $\hat{\sigma}^2_{gc}$, such as:

$$\hat{\mu}_{gc} = \bar{y}_{gc\cdot} = \frac{\sum_{r=1}^{n_c} y_{gcr}}{n_c}$$

where n_c is the sample size of group c.
For heteroscedastic tests, the variance is condition specific :

$$\hat{\sigma}^2_{gc} = S^2_{gc} = \frac{\sum_{r=1}^{n_c} (y_{gcr} - \bar{y}_{gc.})^2}{(n_c - 1)}$$

while homoscedastic tests make the assumption that both groups share a common variance $\hat{\sigma}^2_g$ given by:

$$\hat{\sigma}^2_g = S^2_g = \frac{(n_1 - 1)S^2_{g1} + (n_2 - 1)S^2_{g2}}{n_1 + n_2 - 2}$$

where S^2_g denotes the unbiased pooled estimate of the variance.

Welch's *t*-test
The t-test is a fundamental and commonly applied test in statistics and numerous tests described later

are inspired of it. For any given gene, the test statistic corresponds to a normalized difference between the means of expression levels in both groups:

$$t_g^{\text{Welch}} = \frac{\bar{y}_{g1\cdot} - \bar{y}_{g2\cdot}}{\sqrt{\frac{S_{g1}^2}{n_1} + \frac{S_{g2}^2}{n_2}}}$$

where n_1 and n_2 are number of replicates of group 1 and 2 and $\bar{y}_{g1\cdot}$ is the average expression level for gene g and group 1 (across all possible replicates). Unlike the Student approach, the Welch's t-statistic does not assume equal variances across each group (heteroscedastic hypothesis). The variances S_{g1}^2 and S_{g2}^2 are then estimated independently in both groups for each gene.

The probability of getting the observed t_g^{Welch} value under the null hypothesis is calculated using the Student distribution.

ANOVA (Kerr et al. 2000)
The ANalysis Of VAriance simply consists in studying the relationship between the within-groups variance ($\text{MS}^{\text{within}}$) and the between-groups variance ($\text{MS}^{\text{between}}$). MS denotes the Mean Square, i.e. the Sum of Squares (SS) divided by its degrees of freedom. When there are only two groups of sample:

$$\begin{cases} \text{MS}_g^{\text{between}} = \text{SS}_g^{\text{between}} \\ \text{MS}_g^{\text{within}} = \text{SS}_g^{\text{within}}/(n_1 + n_2 - 2) \end{cases}$$

The general idea behind the ANOVA is simple: if the means of expression levels in both groups are different, then the variance within the groups must be small compared to the variance between the groups.

Therefore, the test statistic consists in the ratio of variances between and within groups, called the F-statistic:

$$F_g = \frac{\text{MS}_g^{\text{between}}}{\text{MS}_g^{\text{within}}}$$

After some algebra, it can be shown that the ANOVA is equivalent to the Student's t-test for two groups:

$$\begin{aligned} \text{SS}_g^{\text{between}} &= \sum_{c=1}^{2} n_c \bar{y}_{gc\cdot}^2 - (n_1 + n_2)\bar{y} \\ &= \frac{(\bar{y}_{g1\cdot} - \bar{y}_{g2\cdot})^2}{\frac{1}{n_1} + \frac{1}{n_2}} \end{aligned}$$

and

$$\begin{aligned} \text{SS}_g^{\text{within}} &= \sum_{c=1}^{2} \sum_{r=1}^{n_c} (y_{gcr} - \bar{y}_{gc\cdot})^2 \\ &= S_g^2 \end{aligned}$$

where $\bar{y}_{gc\cdot}$ is the average expression level for gene g and group c (across all possible replicates). In other words, the F-statistic from the ANOVA is equivalent to the square of the t-statistic of Student for two groups:

$$F_g = \frac{(\bar{y}_{g1\cdot} - \bar{y}_{g2\cdot})^2}{S_g^2(\frac{1}{n_1} + \frac{1}{n_2})}$$

The probability of getting the observed F-statistic under the null hypothesis is calculated using the Fisher

distribution.

RVM (Wright and Simon 2003)
In the RVM model, it is assumed that the gene-specific variances S_g^2 are random variables with an inverse Gamma distribution, whose shape and scale parameters are denoted by a and b. The estimation of both parameters is performed only once for the entire data set by using a maximum likelihood approach. Indeed, Wright and Simon show they can estimate a and b by fitting an F-distribution to the empirical estimates of the gene specific variance (for more details, see Wright et al. [3]).
The statistic is described as follows:

$$t_g^{\mathrm{RVM}} = \frac{\bar{y}_{g1\cdot} - \bar{y}_{g2\cdot}}{S_g^{\mathrm{RVM}}\sqrt{\frac{1}{n_1} + \frac{1}{n_2}}}$$

The variance is given by:

$$(S_g^{\mathrm{RVM}})^2 = \frac{(n_1 + n_2 - 2)S_g^2 + 2a(ab)^{-1}}{(n_1 + n_2 - 2) + 2a}$$

It consists in a weighted average of (i) the pooled variance S_g^2 (with $(n_1 + n_2 - 2)$ degrees of freedom) and (ii) the mean of the fitted inverse gamma distribution $(ab)^{-1}$ (with $2a$ degrees of freedom).
The probability of getting the observed t_g^{RVM} value under the null hypothesis is calculated using the Student distribution.

Limma (Smyth 2004)
The basic statistic used for significance analysis is a moderated t-statistic defined by:

$$t_g^{\mathrm{limma}} = \frac{\bar{y}_{g1\cdot} - \bar{y}_{g2\cdot}}{S_g^{\mathrm{limma}}\sqrt{\frac{1}{n_1} + \frac{1}{n_2}}}$$

This has the same interpretation as an ordinary t-statistic except that the standard errors have been moderated across genes. Indeed, posterior variance, S_g^{limma}, has been substituted into the classical t-statistic in place of the usual variance. Using Bayes rules, this posterior variance becomes a combination of an estimate obtained from the prior distribution (S_0^2) and the pooled variance (S_g^2):

$$S_g^{\mathrm{limma}} = \frac{d_0 S_0^2 + d_g S_g^2}{d_0 + d_g}$$

where d_0 and d_g are, respectively, prior and empirical degrees of freedom. Including a prior distribution on variances has the effect of borrowing information from the ensemble of the genes to aid with inference about each individual gene. Thus, the posterior values shrink the observed variances towards the prior values, that is why it is called "moderated" t-statistic. The probability of getting the observed t_g^{limma} value under the null hypothesis is calculated using the Student distribution.

VarMixt (Delmar et al. 2005)
VarMixt model relies on the assumption that groups of genes can be identified based on similar response to the various sources of variability. The variance of each gene group can be accurately estimated from a large number of observations. Using the group variance in place of individual gene variance, for a given gene the statistic is given by the following expression:

$$t_g^{\mathrm{VM}} = \frac{\bar{y}_{g1\cdot} - \bar{y}_{g2\cdot}}{S_g^{\mathrm{VM}}\sqrt{\frac{1}{n_1} + \frac{1}{n_2}}}$$

Each gene is partially assigned to variance groups: the variance is then a weighted sum of the variance of all the groups:

$$S_g^{\mathrm{VM}} = \sum_1^k \pi_{gi} S_{G_i}^2$$

$G_1, G_2, ..., G_k$ denote the k variance groups of the model. The weight π_{gi} is the posterior probability that the true variance of gene g is $S_{G_i}^2$.
Delmar et al. use an EM approach to determine the number of groups and their associated variances ($S_{G_i}^2$).
The probability of getting the observed t_g^{VM} value under the null hypothesis is calculated using the standard Gaussian distribution.

SMVar (Jaffrezic et al. 2007)
SMVar is an heteroscedastic test, whose test statistic is:

$$t_g^{\mathrm{SMVar}} = \frac{\bar{y}_{g1\cdot} - \bar{y}_{g2\cdot}}{\sqrt{\frac{S_{g1}^2}{n_1} + \frac{S_{g2}^2}{n_2}}}$$

S_{g1}^2 and S_{g2}^2 are estimations of the variance under the following structural model:

$$\ln(S_{gc}^2) = m_c + \delta_{gc}$$

where m_c is a group effect (assumed fixed) and δ_{gc} is the gene effect in a given group c, assumed independent and normally distributed: $\delta_{gc} \sim (0, \tau_c^2)$.

Such model usually requires the use of stochastic estimation procedures based on MCMC methods such as Gibbs sampling that are quite time-consuming. So, Jaffrezic et al. propose an approximate method to obtain estimates of the parameters, based on the empirical variances.
The probability of getting the observed t_g^{SMVar} value under the null hypothesis is calculated using the Student distribution.

1.2 Non-parametric tests

Wilcoxon's test
This test involves the calculation of a statistic, W_g, based on rank summation.
The general process is to combine expression levels from each of two independent groups of observations, listing them in rank order (an average rank is assigned for ties). Then, the rank sums are used to calculate the W_g statistic:

$$W_g = R_{g1} - \frac{(n_1(n_1+1))}{2}$$

where n_1 is the sample size for group 1 and R_{g1} is the rank sums in the same sample.
The idea is that ranks should be randomly arranged between the two groups if observations are drawn from the same underlying distribution.
W_g is then compared to a table of all possible distributions of ranks to calculate the p-value.

SAM (Tusher et al. 2001)
Like the t-test, the purpose of SAM is to express the difference between means in units of standard deviations:

$$t_g^{\mathrm{SAM}} = \frac{\bar{y}_{g1\cdot} - \bar{y}_{g2\cdot}}{S_g\sqrt{\frac{1}{n_1} + \frac{1}{n_2}} + s_0}$$

where S_g is the pooled estimate of the standard deviation, as defined above.
The term s_0 is a "fudge" factor: its purpose is to prevent the statistic t_g^{SAM} from becoming too large when the variance is close to zero (which can lead to false positives). The value of s_0 is some constant performed only once for the entire data. Tusher et al. have initially proposed to determined this factor by a complex procedure based on minimizing the coefficient of variation of t_g^{SAM}. Thereafter, simplified versions have been proposed. For instance, s_0 has been computed as the 90th percentile of the standard errors of all genes.
The probability of getting the observed t_g^{SAM} value under the null hypothesis is computed by Monte-Carlo by permuting the group labels of each observation. In order to obtain an empirical distribution under H_0, t_g^{SAM} is then compared to this empirical distribution to calculate the p-value.

2 Gene list analysis

An intuitive first step to compare the tests is to investigate the consistency between gene lists resulting from the application of each test on real data. Here we apply this approach to five publicly available data sets (Table 1) to assess the overlap between gene lists and to identify similar behaviors among the variance modeling strategies.

In addition to the eight tests, we define a "control" test that draws for each gene a p-value from a Uniform distribution between 0 and 1. Then, we applied the tests to the five data-sets to identify gene differentially expressed by setting a p-value threshold of 0.05.
From the gene lists we construct a binary matrix which contains in rows the genes identified as differentially expressed at least by one test and in columns the 9 tests to compare (see Table S1).

Once the binary matrix has been obtained, we performed two types of analysis to investigate similarities between gene lists: Hierarchical Clustering and Principal Component Analysis.

2.1 Hierarchical Clustering

From the binary matrix we compute a dissimilarity matrix which accounts for pairwise differences between gene lists. Dissimilarity is assessed by using a binary metric, known as the Jaccard distance. Let G_{11} represents the total number of genes identified as differentially expressed by both the t-test and ANOVA. G_{10} (resp. G_{01}) is the number of genes identified as differentially expressed by the t-test (resp. by the ANOVA) but as not differentially expressed by the ANOVA (resp. as not differentially expressed by the t-test). The Jaccard distance is defined as follows:

$$J = \frac{G_{10} + G_{01}}{G_{11} + G_{10} + G_{01}}$$

From the dissimilarity matrix we performed a Hierarchical Agglomerative Clustering. The algorithm starts with every single gene in a single cluster. Then, in each successive iteration, it agglomerates the closest pair of clusters by satisfying some similarity criteria, until all of the data is in one cluster. Here, we use the Ward's approach as linkage method. It consists in minimizing the sum of squares of any two (hypothetical) clusters that can be formed at each step of the hierarchical algorithm. The results are then represented as binary tree, called dendrogram.

2.2 Principal Component Analysis

PCA is a common technique for finding patterns in data of high dimension and expressing the data in such a way as to highlight their similarities and differences. We performed a Principal Component Analysis (PCA) directly on the binary matrix to identify groups of tests that produce similar gene lists.

We specially look at the correlation circle which provides information about correlation between variables (i.e. between gene lists resulting from tests). Thus, two variables forming a small angle are strongly correlated, while a right angle would mean that they are independent.
