# Supplementary material for: Should We Abandon the t-Test in the Analysis of Gene Expression Microarray Data: A Comparison of Variance Modeling Strategies
Source: PLoS One. 2010 Sep 3;5(9):e12336. doi: 10.1371/journal.pone.0012336 (PMC2933223; doi:10.1371/journal.pone.0012336)
Supplement: Table S1 — Example of binary matrix. For a given test, the genes identified as differentially expressed (“1”) and not differentially expressed (“0”) at a given p-value threshold are reported in the binary matrix. (0.01 MB PDF) [file pone.0012336.s002.pdf]

	t-test	ANOVA	Wilcoxon	SAM	RVM	limma	SMVar	VarMixt	Control
**gene 1**	1	1	1	1	1	1	1	1	0
**gene 2**	1	0	0	0	0	0	1	0	0
**gene 3**	1	1	0	0	1	1	1	1	0
